# Are informants required to obtain valid ratings on the Positive and Negative Syndrome Scale (PANSS)?

**DOI:** 10.1038/s41537-023-00378-5

**Published:** 2023-08-31

**Authors:** Cecilie Marie Nielsen, Pernille Kølbæk, David Dines, Mark Opler, Christoph U. Correll, Søren Dinesen Østergaard

**Affiliations:** 1 Psychosis Research Unit, Aarhus University Hospital – Psychiatry, Aarhus, Denmark; 2 Department of Clinical Medicine, Aarhus University, Aarhus, Denmark; 3 Department of Affective Disorders, Aarhus University Hospital – Psychiatry, Aarhus, Denmark; 4 MedAvante-ProPhase Inc, New York, NY USA; 5 Department of Psychiatry, New York University School of Medicine, New York, NY USA; 6 Division of Psychiatry Research, The Zucker Hillside Hospital, Glen Oaks, NY USA; 7 Department of Psychiatry and Molecular Medicine, Donald and Barbara Zucker School of Medicine at Hofstra/Northwell, Hempstead, NY USA; 8 Department of Child and Adolescent Psychiatry and Psychotherapy, Charite Universitätsmedizin, Berlin, Germany

**Keywords:** Schizophrenia, Psychosis

## Abstract

Ratings on the Positive and Negative Syndrome Scale (PANSS) are ideally based on both a patient interview and an informant questionnaire. In research and clinical settings, however, the informant questionnaire is often omitted. This study investigated the consequences of omitting informant information by comparing PANSS ratings of patients with schizophrenia (*n* = 49 patients, 77 ratings) conducted with and without informant information, respectively. Additionally, changes in symptom severity over time based on ratings with and without informant information were also compared for the full PANSS and the six-item version of the PANSS (PANSS-6). PANSS ratings including informant information were higher than those without, both at the total score and individual item level. Additionally, the full PANSS appeared less “responsive” to baseline-to-endpoint changes for ratings without informant information compared to ratings including informant information, while no differences were found for the PANSS-6.

## Introduction

In the treatment of schizophrenia, the use of rating scales to monitor symptom severity over time is likely to lead to improved outcomes^1^. Most schizophrenia symptom severity rating scales are clinician-rated, meaning that the clinician assesses symptoms based on an interview and observations of the patient. However, informants (sometimes referred to as proxies), such as family members or staff members on psychiatric wards, also represent an important source of information for symptom rating, especially when patients lack insight into their illness and symptomatology.

When using the 30-item Positive and Negative Syndrome Scale (PANSS)^2^, often considered to represent the gold standard measure of symptom severity in schizophrenia, two sources of information should be used for ratings: i) a semi-structured interview of the patient, ideally the Structured Clinical Interview for the PANSS (SCI-PANSS)^3^, and ii) information provided by an informant, ideally based on the Informant Questionnaire for the PANSS (IQ-PANSS)^4^. The informant is typically a family member, a close friend, or, if the patient is hospitalized, a staff member. In assessments of behavioral or social impairment, such as passive social withdrawal, the informant should be considered the essential source of information, and, therefore, according to the criteria for PANSS rating, certain items are to be rated solely based on informant information^2^. As the reference period for PANSS ratings is the week prior to the interview, the informant must have been in contact with the patient during this week to provide relevant information. Observations from informants for the week prior to the interview, which may otherwise not be available for the rater, may be required for valid rating on the PANSS.

In both research and clinical settings, there are, however, a number of factors, which may preclude the use of informants for PANSS ratings (e.g., a lack of social network, opposition from the patient, time restraints). In fact, in many clinical trials, including informants is either not part of the protocol, or information on whether informants have contributed to ratings is not provided when the results are published^5–9^. When no informant is available, all PANSS items should be rated on the basis of the clinical semi-structured interview (i.e., the patient’s responses during the interview and the interviewer’s observations) and any other available information^10^. However, it remains unclear how the absence of informant information affects PANSS ratings. To our knowledge, this issue has not been directly addressed previously. Therefore, the present study aimed to compare PANSS ratings that did or did not include information from informants. Specifically, the following research questions were addressed:

i. Are there differences between PANSS scores obtained based on rating of the SCI-PANSS alone (i.e., only interview data) and based on rating of the SCI-PANSS + IQ-PANSS (i.e., including informant data)?
ii. How do scores based on rating of the SCI-PANSS (i.e., only interview data) compare to scores based on rating of the IQ-PANSS (i.e., only informant data)?
iii. Are there differences between baseline-to-endpoint changes on the PANSS when the rating is based only on the SCI-PANSS (i.e., without informant data) and when the rating is based on both the SCI-PANSS + IQ-PANSS (i.e., with informant data)?

## Results

### Patients

Table 1 lists the clinical and sociodemographic characteristics of the 49 participants providing data for the analyses in this study. Their median age was 35 years (interquartile interval (IQI) = 26;48), 53% were male, 63% of the participants had a diagnosis of paranoid schizophrenia (diagnostic code: F20.0)^11^ and the remainder were diagnosed with other schizophrenia subtypes.

**Table 1:** Demographic and clinical characteristics of the 49 participants.

	Total (*n* = 49)	Male (*n* = 26)	Female (*n* = 23)
Age in years, Median (IQI)	35 (26;48)	41 (27;48)	31 (25;50)
Diagnosis, *n* (%)			
Paranoid Schizophrenia (F20.0)	31 (63.3%)	17 (65.4%)	14 (60.9%)
Other (F20.X)	18 (36.7%)	9 (34.6%)	9 (39.1%)
Antipsychotics, *n* (%)			
Any antipsychotic	45 (92%)	24 (92%)	21 (91%)
Apriprazole	21 (42.9%)	8 (30.8%)	13 (56.5%)
Quetiapine	19 (38.8%)	12 (46.2%)	7 (30.4%)
Clozapine	10 (20.4%)	<5^a^	<5^a^
Chlorprothixene	7 (14.3%)	<5^a^	<5^a^
Other	15 (30.6%)	<5^a^	<5^a^
Antidepressants, *n* (%)			
Any antidepressant	22 (44.9%)	11 (42.3%)	11 (47.8%)
SSRI	14 (28.6%)	8 (30.8%)	6 (26.1%)
Other	9 (18.4%)	<5^a^	<5^a^
Antiepileptics, *n* (%)			
Any antiepileptic drug	12 (24.5%)	<5^a^	<5^a^
Drugs for insomnia, *n* (%)			
Benzodiazepines	6 (12.2%)	<5^a^	<5^a^
Z-drugs^b^	7 (14.3%)	<5^a^	<5^a^
Stimulants, *n* (%)			
Central stimulants or Atomoxetine, *n* (%)	<5^a^	<5^a^	<5^a^

IQI: interquartile interval (25th percentile and 75th percentile); Information regarding medication also covers pro re nata use.

^a^Specific numbers cannot be reported due to risk of identification of individual patients.

^b^Zolpidem and Zopiclone.

### Informants

For 56% of the 77 ratings, the informant had either a family or personal relationship with the patient (partner (27%), parent (18%), or friend/other personal relationship (11%)), and for the remaining 44%, the informant had a professional relationship with the patient (case manager (27%) or social worker (17%)).

### Comparison of PANSS ratings based on the SCI-PANSS only versus ratings of the SCI-PANSS + IQ-PANSS

Table 2 lists the results of the comparison between ratings based on the SCI-PANSS alone (i.e., interview data) and ratings based on the SCI-PANSS + IQ-PANSS (i.e., including informant data) for all PANSS items, the sum of the items (PANSS total score), and the three PANSS subscales. Compared to ratings based on the SCI-PANSS ratings alone, ratings based on the SCI-PANSS + IQ-PANSS ratings were statistically significantly higher for 10 out of the 12 items, that should be rated partially based on informant data according to the PANSS instructions. The differences for item P5 – Grandiosity (*z* = 2.236, *p* = 0.063) and item G14 – Poor impulse control (*z* = 2.116, *p* = 0.055) were not statistically significant. Ratings based on the SCI-PANSS + IQ-PANSS were also statistically significantly higher than ratings based on the SCI-PANSS alone for the two items that should be rated solely based on informant data according to the PANSS instructions, namely, item N4 – Passive/apathetic social withdrawal (*z* = 2.259, *p* = 0.023) and item G16 – Active social avoidance (*z* = 2.676, *p* = 0.007). Notably, the PANSS total scores based on ratings of the SCI-PANSS + IQ-PANSS were also statistically significantly higher than those based on ratings of the SCI-PANSS alone (median=63, IQI = 55;74 versus median = 58, IQI = 51;67, *z* = 6.066, *p* = <0.001), as were the sum scores for the positive symptom subscale (median = 17, IQI = 14;20 versus median = 15, IQI = 12;18, *z* = 6.274, *p* = <0.001) and the general psychopathology subscale (median = 31, IQI = 26;36 versus median = 29, IQI = 25;33, *z* = 5.030, *p* = <0.001), but not for the negative symptom subscale (median = 13, IQI = 11;19 versus median = 14, IQI = 11;17, *z* = 0.695, *p* = 0.490). However, for the two items from the negative subscale in which informant information is considered, the statistically significant difference was present (N2 - Emotional withdrawal and N4 - Passive/apathetic social withdrawal).

**Table 2:** PANSS ratings based on i) patient interview only (SCI-PANSS) versus ii) patient interview + informant information (SCI-PANSS + IQ-PANSS) (*n* = 77)^a^.

PANSS items	SCI-PANSS Median (IQI)	SCI-PANSS Mean (SD)	SCI-PANSS + IQ- PANSS Median (IQI)	SCI-PANSS + IQ-PANSS Mean (SD)	*Z*-value	*P*-value (Wilcoxon Signed Rank test)
**Delusions (P1)**	4 (2;4)	3.3 (1.6)	4 (2;5)	3.6 (1.7)	3.461	<0.001*
Conceptual disorganization (P2)	1 (1;2)	1.7 (1.1)	1 (1;2)	1.7 (1.1)	–	1.000
**Hallucinatory behavior (P3)**	4 (2;5)	3.5 (1.6)	4 (2;5)	3.7 (1.7)	2.999	0.004*
**Excitement (P4)**	1 (1;1)	1.5 (0.9)	1 (1;3)	2.3 (1.8)	5.067	<0.001*
**Grandiosity (P5)**	1 (1;1)	1.5 (1.0)	1 (1;2)	1.7 (1.3)	2.236	0.063
**Suspiciousness/persecution (P6)**	3 (1;4)	2.8 (1.5)	3 (1;4)	3.1 (1.7)	2.904	0.004*
**Hostility (P7)**	1 (1;1)	1.2 (0.5)	1 (1;1)	1.5 (1.1)	3.461	<0.001*
Blunted affect (N1)	3 (2;4)	3.0 (1.4)	3 (2;4)	3.0 (1.4)	–	1.000
**Emotional withdrawal (N2)**	2 (1;3)	2.3 (1.2)	3 (1;4)	2.7 (1.6)	3.761	<0.001*
Poor rapport (N3)	1 (1;2)	1.6 (1.0)	1 (1;2)	1.6 (1.0)	–	1.000
**Passive/apathetic social withdrawal (N4)**	3 (1;3)	2.5 (1.4)	2 (1;5)	2.9 (2.1)	2.259	0.023*
Difficulty in abstract thinking (N5)	2 (1;3)	2.1 (1.3)	2 (1;3)	2.1 (1.3)	–	1.000
Lack of spontaneity and flow of conversation (N6)	1 (1;2)	1.8 (1.0)	1 (1;2)	1.8 (1.0)	–	1.000
Stereotyped thinking (N7)	1 (1;1)	1.3 (0.6)	1 (1;1)	1.3 (0.6)	–	1.000
Somatic concern (G1)	1 (1;2)	1.8 (1.3)	1 (1;2)	1.8 (1.3)	–	1.000
Anxiety (G2)	4 (3;5)	3.7 (1.7)	4 (3;5)	3.7 (1.7)	–	1.000
Guilt feelings (G3)	2 (1;4)	2.5 (1.7)	2 (1;4)	2.5 (1.7)	–	1.000
Tension (G4)	1 (1;2)	1.7 (1.0)	1 (1;2)	1.7 (1.0)	–	1.000
**Mannerisms and posturing (G5)**	1 (1;1)	1.2 (0.6)	1 (1;1)	1.4 (0.8)	2.449	0.031*
**Depression (G6)**	3 (3;4)	3.4 (1.5)	4 (3;5)	3.7 (1.6)	3.461	0.001*
**Motor retardation (G7)**	1 (1;2)	1.7 (1.0)	1 (1;3)	2.0 (1.4)	3.047	0.002*
**Uncooperativeness (G8)**	1 (1;1)	1.1 (0.2)	1 (1;1)	1.4 (1.1)	2.645	0.016*
Unusual thought content (G9)	2 (1;3)	2.1 (1.3)	2 (1;3)	2.1 (1.3)	–	1.000
Disorientation (G10)	1 (1;2)	1.6 (0.9)	1 (1;2)	1.6 (0.9)	–	1.000
Poor attention (G11)	1 (1;1)	1.3 (0.6)	1 (1;1)	1.3 (0.6)	–	1.000
Lack of judgment and insight (G12)	2 (1;3)	2.2 (1.3)	2 (1;3)	2.2 (1.3)	–	1.000
Disturbance of volition (G13)	1 (1;1)	1.2 (0.5)	1 (1;1)	1.2 (0.5)	–	1.000
**Poor impulse control (G14)**	1 (1;2)	1.5 (1.3)	1 (1;2)	1.7 (1.1)	2.116	0.055
Preoccupation (G15)	1 (1;1)	1.1 (0.5)	1 (1;1)	1.1 (0.5)	–	1.000
**Active social avoidance (G16)**	1 (1;3)	1.9 (1.3)	1 (1;3)	2.2 (1.8)	2.676	0.007*
**PANSS SUBSCALES/TOTAL SCORE**						
Positive subscale	15 (12;18)	15.4 (4.4)	17 (14;20)	17.5 (5.7)	6.274	<0.001*
Negative subscale	14 (11;17)	14.5 (4.9)	13 (11;19)	14.9 (5.1)	0.695	0.490
General psychopathology subscale	29 (25;33)	30.0 (7.6)	31 (26;36)	31.5 (8.3)	5.030	<0.001*
PANSS total score	58 (51;67)	59.9 (13.8)	63 (55;74)	64.3 (16.0)	6.066	<0.001*

Items in bold are part of the IQ-PANSS. For the 16 items, which are not part of the IQ-PANSS, scores based on the SCI-PANSS + IQ-PANSS and scores based on the SQI-PANSS were identical. When comparing the median PANSS total scores (obtained using both the SCI-PANSS and the IQ-PANSS) of the 28 participants rated both at baseline and endpoint with those of the 21 participants rated only at baseline, we found no statistically significant difference.

*IQI* Interquartile interval (25th and 75th percentile).

*p < 0.05.

^a^49 participants with an informant contributing to the PANSS ratings were included, 28 of whom were rated twice, yielding a total of 77 PANSS ratings (i.e., 49 baseline ratings and 28 endpoint ratings)

### Comparison of ratings based on the SCI-PANSS versus ratings based on the IQ-PANSS

Table 3 lists results of the comparison between ratings based on the SCI-PANSS (i.e., using only interview data) and ratings based on the IQ-PANSS (i.e., using only informant data) for the 14 PANSS items covered by the IQ-PANSS. Ratings based on the SCI-PANSS were statistically significantly higher for items P1 – Delusions, P3 – Hallucinatory behavior, P6 – Suspiciousness/persecution, G6 – Depression, and G14 – Poor impulse control, while ratings based on the IQ-PANSS were statistically significantly higher for item P4 – Excitement.

**Table 3:** PANSS-ratings based on i) patient interview only (SCI-PANSS) versus ii) informant questionnaire only (IQ-PANSS) (*n* = 77)^a^.

	SCI-PANSS Median (IQI)	SCI-PANSS Mean (SD)	IQ-PANSS Median (IQI)	IQ-PANSS Mean (SD)	*Z*-value	*P*-value (Wilcoxon Signed Rank test)
Delusions (P1)	4 (2;4)	3.3 (1.6)	1 (1;4)	2.3 (1.8)	4.110	<0.001*
Hallucinatory behavior (P3)	4 (2;5)	3.5 (1.6)	1 (1;3)	2.6 (1.9)	3.984	<0.001*
Excitement (P4)	1 (1;1)	1.5 (0.9)	1 (1;3)	2.2 (1.8)	−3.370	0.001*
Grandiosity (P5)	1 (1;1)	1.5 (1.0)	1 (1;1)	1.4 (1.1)	1.651	0.113
Suspiciousness/persecution (P6)	3 (1;4)	2.8 (1.5)	1 (1;4)	2.2 (1.7)	3.693	<0.001*
Hostility (P7)	1 (1;1)	1.2 (0.5)	1 (1;1)	1.4 (1.0)	−1.274	0.192
Emotional withdrawal (N2)	2 (1;3)	2.3 (1.2)	1 (1;4)	2.2 (1.7)	0.914	0.367
Passive/apathetic social withdrawal (N4)	3 (1;3)	2.5 (1.4)	1 (1;4)	2.5 (2.2)	0.348	0.732
Mannerisms and posturing (G5)	1 (1;1)	1.2 (0.6)	1 (1;1)	1.2 (0.7)	0.876	0.452
Depression (G6)	3 (3;4)	3.4 (1.5)	3 (1;4)	2.7 (1.8)	3.973	<0.001*
Motor retardation (G7)	1 (1;2)	1.7 (1.0)	1 (1;1)	1.7 (1.4)	0.724	0.475
Uncooperativeness (G8)	1 (1;1)	1.1 (0.2)	1 (1;1)	1.3 (1.1)	−1.021	0.228
Poor impulse control (G14)	1 (1;2)	1.5 (0.9)	1 (1;1)	1.3 (0.9)	2.177	0.029*
Active social avoidance (G16)	1 (1;3)	1.9 (1.3)	1 (1;1)	1.8 (1.7)	1.375	0.170

*IQI* Interquartile interval.

*p < 0.05.

^a^49 participants with an informant contributing to the PANSS ratings were included, 28 of whom were rated twice, yielding a total of 77 PANSS ratings (i.e., 49 baseline ratings and 28 endpoint ratings).

### Comparison of baseline-to-endpoint changes based on ratings of the SCI-PANSS only versus ratings based on the SCI-PANSS + IQ-PANSS

Table 4 lists the results of the comparison of baseline-to-endpoint changes for ratings based on the SCI-PANSS alone and ratings based on the SCI-PANSS + IQ-PANSS combined for all items, the PANSS total score, and the three PANSS subscale scores, respectively. The baseline-to-endpoint change was statistically significantly lower when using ratings based on the SCI-PANSS only compared to ratings based on the SCI-PANSS + IQ-PANSS combined for item G16 – Active social avoidance (*z* = −2.444, *p* = 0.031), for the PANSS total score (median change from baseline to endpoint = −1.5, IQI = −8.5;8.0 versus −3.0, IQI = −9.5;5.5, *z* = −2.161, *p* = 0.030), and for the sum score for the general psychopathology subscale (median change from baseline to endpoint = 0.0, IQI = −5.5;2.0 versus −1.5, IQI = −6.5;2.0, *z* = −2.855, *p* = 0.003).

**Table 4:** Differences in assessment of change from baseline to endpoint of PANSS-ratings based on i) patient interview only (SCI-PANSS) versus ii) both patient interview and informant information (SCI-PANSS + IQ-PANSS) (*n* = 28).

PANSS items	SCI-PANSS Median (IQI)	SCI-PANSS Mean (SD)	SCI-PANSS + IQ-PANSS Median (IQI)	SCI-PANSS + IQ-PANSS Mean (SD)	*Z*-value	*P*-value (Wilcoxon Signed Rank test)
**Delusions (P1)**	0 (−1.0;0.5)	−0.3 (1.4)	0 (−1.0;1.0)	−0.2 (1.9)	0.377	0.797
Conceptual disorganization (P2)	0 (0.0;0.0)	0.0 (0.8)	0 (0.0;0.0)	0.0 (0.8)	–	1.000
**Hallucinatory behavior (P3)**	0 (−1.0;0.0)	−0.1 (0.9)	0 (−1.0;0.0)	−0.4 (1.0)	−1.731	0.250
**Excitement (P4)**	0 (−0.5;0.0)	−0.2 (1.3)	0 (−1.0;0.0)	−0.2 (1.5)	–	1.000
**Grandiosity (P5)**	0 (0.0;1.0)	0.5 (1.0)	0 (0.0;0.5)	0.1 (1.5)	−1.414	0.500
**Suspiciousness/persecution (P6)**	0 (0.0;1.0)	0.1 (1.1)	0 (0.0;1.0)	0.3 (1.3)	0.792	0.445
**Hostility (P7)**	0 (0.0;0.0)	0.1 (0.4)	0 (0.0;0.0)	−0.2 (1.1)	−1.101	0.391
Blunted affect (N1)	0 (−2.0;1.0)	−0.3 (1.9)	0 (−2.0;1.0)	−0.3 (1.9)	–	1.000
**Emotional withdrawal (N2)**	0 (−1.0;1.0)	−0.1 (1.0)	0 (−1.0;1.0)	−0.3 (1.5)	−0.897	0.412
Poor rapport (N3)	0 (0.0;0.0)	0.1 (1.0)	0 (0.0;0.0)	0.1 (1.0)	−	1.000
**Passive/apathetic social withdrawal (N4)**	0 (0.0;1.0)	0.1 (1.4)	0 (0.0;0.0)	0.1 (2.0)	−0.109	0.926
Difficulty in abstract thinking (N5)	1 (0.0;1.5)	0.7 (1.1)	1 (0.0;1.5)	0.7 (1.1)	–	1.000
Lack of spontaneity and flow of conversation (N6)	0 (−1.0;0.5)	−0.2 (1.3)	0 (−1.0;0.5)	−0.2 (1.3)	–	1.000
Stereotyped thinking (N7)	0 (0.0;0.0)	0.0 (0.7)	0 (0.0;0.0)	0.0 (0.7)	–	1.000
Somatic concern (G1)	0 (−1.0;0.0)	−0.6 (1.5)	0 (−1.0;0.0)	−0.6 (1.5)	–	1.000
Anxiety (G2)	0 (0.0;1.0)	0.2 (1.3)	0 (0.0;1.0)	0.2 (1.3)	–	1.000
Guilt feelings (G3)	0 (−0.5;2.0)	0.4 (2.4)	0 (−0.5;2.0)	0.4 (2.4)	–	1.000
Tension (G4)	0 (−1.0;0.0)	−0.4 (1.2)	0 (−1.0;0.0)	−0.4 (1.2)	–	1.000
**Mannerisms and posturing (G5)**	0 (0.0;0.0)	−0.1 (0.8)	0 (−1.0;0.0)	−0.4 (0.8)	−1.999	0.125
**Depression (G6)**	0 (−1.0;1.0)	−0.1 (1.6)	0 (−2.0;0.0)	−0.4 (1.4)	−1.554	0.094
**Motor retardation (G7)**	0 (0.0;1.0)	0.3 (1.2)	0 (0.0;1.0)	0.1 (1.4)	−0.690	0.547
**Uncooperativeness (G8)**	0 (0.0;0.0)	0.0 (0.3)	0 (0.0;0.0)	0.0 (0.7)	−0.026	1.000
Unusual thought content (G9)	0 (−1.0;0.5)	−0.1 (1.5)	0 (−1.0;0.5)	−0.1 (1.5)	–	1.000
Disorientation (G10)	0 (0.0;0.0)	0.0 (0.6)	0 (0.0;0.0)	0.0 (0.6)	–	1.000
Poor attention (G11)	0 (0.0;0.0)	0.0 (0.8)	0 (0.0;0.0)	0.0 (0.8)	–	1.000
Lack of judgement and insight (G12)	0 (0.0;0.0)	0.0 (1.1)	0 (0.0;0.0)	0.0 (1.1)	–	1.000
Disturbance of volition (G13)	0 (0.0;0.5)	0.3 (0.7)	0 (0.0;0.5)	0.3 (0.7)	–	1.000
**Poor impulse control (G14)**	0 (0.0;0.0)	−0.1 (0.9)	0 (0.0;0.0)	0.0 (0.8)	0.038	1.000
Preoccupation (G15)	0 (0.0;0.0)	0.1 (0.3)	0 (0.0;0.0)	0.1 (0.3)	–	1.000
**Active social avoidance (G16)**	0 (−1.0;0.0)	−0.4 (1.3)	0 (−1.5;0.0)	−0.8 (1.1)	−2.444	0.031*
**PANSS SUBSCALES/TOTAL SCORE**						
Positive subscale	0.0 (−2.0;2.0)	0.1 (3.7)	−1.0 (−4.0;2.5)	−0.5 (4.4)	−1.125	0.275
Negative subscale	1.0 (−1.0;3.0)	0.3 (5.0)	0.0 (−2.0;2.5)	0.1 (5.0)	−0.260	0.805
General psychopathology subscale	0.0 (−5.5;2.0)	−0.5 (7.0)	−1.5 (−6.5;2.0)	−1.7 (6.4)	−2.855	0.003*
PANSS total score	−1.5 (−8.5;8.0)	0.0 (12.0)	−3.0 (−9.5;5.5)	−2.0 (11.2)	−2.161	0.030*

Items in bold are part of the IQ-PANSS.

*IQI* Interquartile interval.

*p < 0.05.

The results of the post hoc analysis revealed no material or statistically significant difference in the baseline-to-endpoint change on the PANSS-6 when based on ratings of the SCI-PANSS alone versus when based on ratings of the SCI-PANSS + IQ-PANSS combined (median change from baseline to endpoint = 0.0, IQI = −3.0;1.0 versus −1.0, IQI = −3.0;1.0, *z* = 0.885, *p* = 0.3805).

## Discussion

This study aimed to investigate the consequences of not including informant information during PANSS rating. The results suggest that omitting informant information does, indeed, have consequences that may be important to consider when using the PANSS for research and clinical purposes. Specifically, the answers to the three research questions were as follows: I) PANSS scores based only on ratings of the SCI-PANSS were consistently lower than those based on ratings of both the SCI-PANSS and the IQ-PANSS, both at the item and the total score levels; II) Scores based on the SCI-PANSS were generally higher than scores based on the IQ-PANSS; and III) Changes (reductions predominantly) in symptom severity over time were generally reduced when scores were based only on ratings of the SCI-PANSS compared to when scores were based on ratings of both the SCI-PANSS and the IQ-PANSS. Conversely, a post hoc analysis revealed no material or statistically significant difference in assessment of changes over time in the PANSS-6 total score between ratings based on the SCI-PANSS alone and ratings based on both the SCI-PANSS and the IQ-PANSS.

With regard to the comparison of scores based only on the SCI-PANSS and scores based on the SCI-PANSS + IQ-PANSS, our results indicate that there is an additive effect of using the SCI-PANSS and the IQ-PANSS (i.e., adding up information from both sources results in higher scores) for almost all PANSS items that are to be rated based on both the SCI-PANSS and the IQ-PANSS. Thus, in certain cases, the criteria for a higher score were only met when adding the information from both sources of information (i.e., ratings based on the clinical semi-structured interview and information provided by an informant), and not by either source alone. This finding strongly suggests that the raters adhered to one of the basic principles for conducting PANSS ratings, namely, to assign the highest score for which the criteria are met^2,10^. Notably, however, we also found that the mean scores based on ratings of the SCI-PANSS + IQ-PANSS were higher than those based only on ratings of the SCI-PANSS for items N4 **–** Passive/apathetic social withdrawal and G16 **–** Active social avoidance, which, according to the instructions, are to be rated solely based on the IQ-PANSS. Thus, these results indicate that, for these two items, raters did not always adhere to the instructions but rather used information from both the IQ-PANSS and the SCI-PANSS for their assessments. In the publication introducing the PANSS, Kay et al. point out that informants should generally be considered as the essential source of information for the rating of items pertaining to social impairment^2^. However, according to the specific item-level instructions for several items related to social impairment, for example, P7 **–** Hostility and G14 **–** Poor impulse control, ratings are to be based on both the SCI-PANSS and IQ-PANSS, suggesting that both sources are considered relevant for these items. According to our results, it seems that the SCI-PANSS also provides relevant information for items N4 **–** Passive/apathetic social withdrawal and G16 **–** Active social avoidance. Intuitively, it appears meaningful that the patient interview and the associated observation of the patient may provide valid information with regard to the assessment of these two symptom items, and it may therefore be relevant to revise the criteria for rating accordingly. This approach would be in line with the basic PANSS principle of using all relevant information for ratings and would also have the benefit of streamlining the rating principles for all items that are part of the IQ-PANSS.

As all PANSS scores at the item level were higher when based on ratings of both the SCI-PANSS and IQ-PANSS compared to when based only on ratings of the SCI-PANSS, a mean difference of 4.3 points between the PANSS total scores was observed, corresponding to a 14.7% higher score (when calculating the percent change for the PANSS, a subtraction of 30 points from the total score is necessary as the minimum score is 30 due to the Likert scale range of 1–7 for each item)^12,13^. If PANSS scores obtained via ratings of both the SCI-PANSS and the IQ-PANSS are considered the gold standard reference, this suggests that using only the SCI-PANSS leads to a substantial measurement error. Notably, the difference due to factoring in the IQ-PANSS was evident although scores based on ratings of the IQ-PANSS alone were substantially lower than those obtained based on ratings of the SCI-PANSS alone, which is consistent with our previous findings^14^. At the subscale level, unlike the positive symptom and the general psychopathology subscales, which followed the pattern for the full PANSS, statistically significant differences were not found when comparing ratings on the negative symptom subscale based on the SCI-PANSS alone to those based on both the IQ-PANSS and the SCI-PANSS. This finding may be explained by the fact that informant data are only to be used for scoring two out of seven items of the negative symptom subscale.

In clinical trials in schizophrenia, the PANSS is commonly used as the primary outcome measure^15–17^. This use relies entirely on the PANSS being sensitive to changes in symptom severity over time, due to, e.g., psychopharmacological treatment. In this study, we found that sensitivity to symptom change according to the PANSS appears to be reduced when informant data was not factored in (i.e., when scores were based on ratings of the SCI-PANSS alone). This result was evident at the level of the PANSS total score and appeared to be driven primarily by the general psychopathology subscale score. In this context, it is noteworthy that when Kay et al. introduced the PANSS, the general psychopathology subscale was merely presented as an adjunct to the positive and negative symptom subscale, and was not meant to be included in the PANSS total score, as it is not particularly informative with regard to the core symptoms of schizophrenia^2^. Subsequently, the field has tended to include the general psychopathology subscale score in the PANSS total score^17^. According to the results of the present study, this inclusion of the general psychopathology subscale may have come at the cost of the PANSS being less sensitive to change when not including informant data. In accordance with this finding, when conducting ratings on the PANSS-6, which contains no items from the general psychopathology subscale, the sensitivity to change was, seemingly, not affected by not factoring in informant data.

Based on our experience from using the IQ-PANSS in the study that provided data for the present study^18^ as well as from a similar study conducted among inpatients^19^, many informants find the IQ-PANSS rather difficult to complete due to both the structure/layout of the questionnaire and the terminology that is used. Consequently, relevant information may not be reported, which may partially explain some of the differences in scores based on the IQ-PANSS and the SCI-PANSS. Therefore, it seems advisable to consider a revision of the IQ-PANSS to make it more accessible to informants. In this context, it may also be relevant to consider using an interview instead of a questionnaire for the collection of informant information. An interview may allow for a more flexible approach, enabling the interviewer to ask clarifying questions, ensure that the correct reference period is used, and, if necessary, to explain unfamiliar terms related to psychopathology to the informant. Furthermore, an interview can be conducted over the phone, which may make informant involvement more feasible.

In addition to the relatively small sample size, a number of limitations must be considered when interpreting the results of the present study. First and foremost, PANSS ratings without informant data were made only based on the SCI-PANSS (i.e., the patient interview). Ideally, when an informant is not available, additional sources of information, for example, electronic health records, can supplement the patient interview^10^. The availability of such supplemental information could have led to less pronounced differences between the PANSS scores obtained with and without informant data, respectively, in the present study. However, in clinical trials, this type of information may not be available either. Second and relatedly, according to the PANSS-guidelines, informants should be contacted whenever further elaboration or clarification is necessary^4^. This procedure was not followed in the present study. Third, the informants’ understanding of and adherence to the IQ-PANSS (e.g., their understanding of psychopathology and whether they based their assessment on the past week) and, hence, the validity of the information they provided was not checked or verified. However, the informants were provided with a set of instructions to facilitate assessment and were encouraged to contact the first author if they had any questions related to the completion of the IQ-PANSS. These instructions might have introduced some bias/variation, which should be taken into account when comparing the results of this study with those of future studies employing a different approach with regard to the IQ-PANSS. Fourth, the PANSS ratings based on the SCI-PANSS and the PANSS ratings based on the IQ-PANSS were conducted by the same rater, and, as a rule, the SCI-PANSS-based rating was completed first. This procedure might have caused some bias in the PANSS ratings based on the IQ-PANSS (e.g., a spillover effect). If so, this bias would most likely have tended towards a better agreement between the PANSS ratings based on the patient interview and the informant data, which diverged, nevertheless. Fifth, the inter-rater reliability of the raters was not examined with regard to their PANSS ratings based on the IQ-PANSS, specifically. The general principles for ratings based on the IQ-PANSS are, however, the same as those used for ratings based on the SCI-PANSS (where the inter-rater reliability of the raters was confirmed). Sixth, the participating patients were rather young. As information from informants is likely particularly important for valid ratings of older patients, due to, e.g., cognitive impairment and illness chronicity, we may have underestimated the consequences of PANSS rating without informants. Seventh, the study providing data for the analyses in this paper only included outpatients with schizophrenia. Inpatients and outpatients with schizophrenia differ in relation to clinically important measures, including symptom severity, compliance, and insight^20^. Therefore, the results from this study cannot necessarily be generalized to inpatient populations. Future studies should therefore aim to examine how the absence of data provided by informants affects PANSS ratings of inpatients.

In conclusion, this study revealed substantial and statistically significant differences in PANSS scores obtained based on ratings of the SCI-PANSS alone (i.e., without informant data) and based on ratings of both the SCI-PANSS and the IQ-PANSS (i.e., including informant data). The same differences were observed in relation to the assessment of change in PANSS scores over time, where the assessment without informant data appeared less sensitive to change. Notably, the sensitivity to change of the PANSS-6 may be less affected by the lack of informant data. Although preliminary, these results suggest that PANSS scores obtained with and without informant data, respectively, may not be comparable for the same patient over time, between patients or between studies. While these findings need replication via other studies, they suggest that differences in PANSS ratings obtained with and without informant data, respectively, should be taken into account when using the PANSS for both research and clinical purposes.

## Methods

### Participants

This study is based on data from Nielsen et al.^18^, which aimed to validate the six-item version of the PANSS (PANSS-6) by using ratings on the full PANSS, which were derived from the SCI-PANSS and the IQ-PANSS, as the gold standard reference. Seventy-three patients with schizophrenia attending the psychiatric outpatient clinic at Aarhus University Hospital in Denmark participated in this study. Patients were eligible to participate if they were at least 18 years old, met the International Classification of Disease, 10th edition (ICD-10) criteria for schizophrenia according to the treating psychiatrist, understood written and spoken Danish, and provided written informed consent. Patients were ineligible for participation if they were admitted to a psychiatric inpatient unit, had an organic mental disorder, mental retardation, or were under the influence of any substances of abuse, including alcohol (as per clinical judgement by the treating psychiatrist).

Whenever possible, an informant contributed to the PANSS ratings. This was feasible for 49 participants, among whom 28 were rated twice (at least 2 months apart), yielding a total of 77 PANSS ratings (49 baseline ratings and 28 endpoint ratings), which defined the dataset analyzed in this study.

### Measures

The PANSS contains 30 items that cover three subscales: a 7-item positive symptom subscale, a 7-item negative symptom subscale and a 16-item general psychopathology subscale^2^. Ideally, the information for rating the PANSS is obtained using the SCI-PANSS (a semi-structured patient interview) and the IQ-PANSS^3,4^. The IQ-PANSS was designed to streamline the process for obtaining informant information^4^ and contains questions related to the following 14 PANSS items, which, according to the rating criteria for the PANSS^2^, require informant information for optimal rating: P1 – Delusions, P3 – Hallucinatory behavior, P4 – Excitement, P5 – Grandiosity, P6 – Suspiciousness/persecution, P7 – Hostility, N2 – Emotional withdrawal, N4 – Passive/apathetic social withdrawal, G5 – Mannerisms and posturing, G6 – Depression, G7 – Motor retardation, G8 – Uncooperativeness, G14 – Poor impulse control, and G16 – Active social avoidance^4^. Of these 14 items, items N4 – Passive/apathetic social withdrawal and G16 – Active social avoidance, are to be rated solely based on the IQ-PANSS. When completing the IQ-PANSS, informants respond “yes” or “no” to a number of questions related to each of the 14 items. Furthermore, for each item, there is a designated free text space, in which informants are encouraged to specify any observed behavior related to the item^4^.

We developed a set of written instructions to accompany the IQ-PANSS to aid informants, especially if they did not have clinical experience (e.g., the patients' relatives or friends). These additional instructions included a general introduction on how to fill out the IQ-PANSS (e.g., “In the following questionnaire you will be presented to some of the symptoms that can be observed among people with schizophrenia. Please indicate whether the specific symptoms, as they are described in the text, have been evident during the last week.”) as well as explanations of various terms describing psychopathology, which may not be familiar to laypeople (e.g., “Hallucinations are sensory perceptions that seem real to the patient, but are not caused by an external source, and therefore cannot be seen, heard, smelled, tasted or felt by others. They may be voices, sounds or visions, that others cannot hear or see”). An English translation of these instructions is provided in the Supplementary Material.

### Procedure

Two certified PANSS raters (a nurse and a psychologist) conducted all the SCI-PANSS interviews. These interviews were videotaped and subsequently rated by a team of four certified PANSS raters (one psychiatrist, one psychiatric resident, and two final year medical students) with established inter-rater reliability (Intra class coefficient for the PANSS total score: 0.85, 95% CI 0.70–0.95). For further information on the certification, training, and inter-rater reliability of the raters, see Nielsen et al.^18^. The informants completed either a paper or digital version of the IQ-PANSS, and were encouraged to contact the first author (CMN) if they had any questions regarding the IQ-PANSS.

The same rater conducted the PANSS ratings of the SCI-PANSS interview and thereafter interpreted and rated the IQ-PANSS. Specifically, the raters' instructions were as follows: i) rate all 30 PANSS items based solely on the information derived from the SCI-PANSS, then ii) rate the 14 items included in the IQ-PANSS based solely on the information derived from the IQ-PANSS; and finally iii) rate all 30 items using both sources of information (i.e., the SCI-PANSS and the IQ-PANSS). In the final rating, raters were instructed to adhere to the PANSS instructions that specify which information should be used as the basis for the rating of each item (i.e., information from the SCI-PANSS, the IQ-PANSS, or both sources). The instructions for the IQ-PANSS specify that informants should be contacted if further elaboration or clarification is necessary. However, for logistical reasons, this procedure was not followed in this study.

### Statistical analyses

The clinical and sociodemographic characteristics of the 49 participants were reported using descriptive statistics. As a substantial proportion of the data was not normally distributed, the nonparametric Wilcoxon Signed Rank Test was used for all the comparisons. Ratings based on the SCI-PANSS alone (i.e., only interview data) and ratings based on the SCI-PANSS + IQ-PANSS (i.e., including informant data) were compared for all individual PANSS items, as well as for the total score and the three subscale scores (positive, negative, and general psychopathology subscales). Additionally, ratings based on the SCI-PANSS (i.e., without informant data) and ratings based on the IQ-PANSS (i.e., only using informant data) were compared for all individual PANSS items, as well as for the total score and subscale scores. Finally, the changes from baseline-to-endpoint according to ratings of the SCI-PANSS alone and ratings of the SCI-PANSS + IQ-PANSS were compared for all individual PANSS items, as well as for the total score and the three subscale scores. The comparisons of changes were based on data from the subset of participants with both a baseline and an endpoint rating (*n* = 28), and the changes in scores based on ratings of the SCI-PANSS + IQ-PANSS were used as the reference. All analyses were two-tailed, conducted using Stata v17.0 (StataCorp, College Station, TX) and with alpha = 0.05.

### Post hoc analysis

Motivated by the results of the comparisons of baseline-to-endpoint changes based on ratings using the SCI-PANSS alone and ratings of the SCI-PANSS + IQ-PANSS combined, an analogous post-hoc comparison of baseline-to-endpoint changes in the total score of the six-item version of the PANSS (PANSS-6)^21^ was conducted. The PANSS-6, which consists of the items P1 *–* Delusions, P2 *–* Conceptual disorganization, P3 *–* Hallucinations, N1 *–* Blunted affect, N4 *–* Passive/apathetic social withdrawal, and N6 *–* Lack of spontaneity and flow in conversations, is a brief alternative to the full PANSS and has shown promising psychometric properties^19,21–24^.

### Ethics

Written informed consent was obtained from all participants. For further details, see Nielsen et al.^18^. All data were processed and stored in accordance with the European Union General Data Protection Regulation.

### Supplementary information

Supplementary Material

## Data Availability

The consent of the participants does not allow for data sharing.
