# Supplementary material for: Are informants required to obtain valid ratings on the Positive and Negative Syndrome Scale (PANSS)?
Source: Schizophrenia (Heidelb). 2023 Aug 31;9(1):54. doi: 10.1038/s41537-023-00378-5 (PMC10471695; doi:10.1038/s41537-023-00378-5)
Supplement: Supplementary file 1 — Supplementary Material [file 41537_2023_378_MOESM1_ESM.docx]

**Supplementary Material**

Written instructions, developed for the study by Nielsen et al.^1^ to accompany the IQ-PANSS. The instructions include a general introduction (A), and explanations related to specific items (B).

**A. Questionnaire regarding symptoms of schizophrenia**

**DIRECTIONS FOR THE QUESTIONNAIRE:**

In the following questionnaire you will be presented to some of the symptoms that can be observed among people with schizophrenia. Please indicate whether the specific symptoms, as they are described in the text, have been evident ***during the last week.*** In this questionnaire we will refer to your relative as 'the patient'.

Please pay particular attention to words and/or phrases that appear in **boldface print**, for example 'and', 'or', or words that indicate something about the necessary frequency of the symptom required to fulfill the criteria.

If a symptom has not been present during the last week, then put a checkmark by **'Symptom is absent'** and move on to the next symptom. The answer category **'Questionable pathology; may be at the upper extreme of normal limits'** is used, when a symptom has been present, but not to a degree that any of the other statements could be checked 'yes'. Under each symptom additional space is provided for specifying the symptoms, which has been evident during the past week.

Throughout the questionnaire some additional explanations or examples have been added in blue print. The instructions that you have just read have been made specifically for this project. The original instructions are inserted on the next page and you can read them if you like, otherwise move on to page 3, where the questionnaire begins.

**B) Explanations related to specific items**

| **Item** | **Subsection** | **Added text** |
| --- | --- | --- |
| P1 | Definition | Examples of delusions are the belief that others can hear or read one's thoughts, to have special abilities or supernatural powers, or to be watched or persecuted. That the beliefs are idiosyncratic means that the belief is not shared by others. |
| P1 | Questions related to a rating of 1 | Put a checkmark here if the symptom, as described above, is not present  (This comment was inserted for every item throughout the questionnaire). |
| P1 | Questions related to a rating of 2 | Put a checkmark here if it is unsure, whether there is pathology  (This comment was inserted for every item throughout the questionnaire). |
| P1 | Questions related to a rating of 2 | ('Pathology' means that something deviates from the normal or the healthy, in other words something that is related to illness.)  (This comment was inserted for every item throughout the questionnaire). |
| P1 | Questions related to a rating of 3 | ('Delusions that are not tenaciously held' means delusions that the patient is not convinced about all the time or that the patient does not hold on to if others challenge them or argues against them.) |
| P1 | Questions related to a rating of 3 | ('Unstable delusions' means delusions that are not there all the time or that change over time.) |
| P1 | Questions related to a rating of 6 | ('A stable set of delusions' means several/more than one delusion which are there all the time and do not change over time) |
| P1 | Questions related to a rating of 6 | ('Systematized delusions' means that the delusions are put in system and are connected to each other.) |
| P1 | Questions related to a rating of 6 | ('Delusions that are tenaciously held' means delusions that the patient is convinced about and holds on to even if others challenge them or argues against them.) |
| P3 | Definition | Hallucinations are sensory perceptions that seem real to the patient, but are not caused by an external source, and therefore cannot be seen, heard, smelled, tasted or felt by others. They may be voices, sounds or visions, that others cannot hear or see. |
| P3 | Questions related to a rating of 5 | ('A sensory modality' means a 'sense for example hearing, sight or smell.) |
| P3 | Questions related to a rating of 5 | ('A delusional interpretation' means an interpretation that is based on a delusion |
| P3 | Questions related to a rating of 5 | ('To respond verbally' means to say something as a reaction to the hallucinations; it can be talking to/with or yelling at the hallucinations, for example to ask them to be quiet.) |
| P3 | Questions related to a rating 5 | ('To respond emotionally' means to have an emotional reaction to the hallucinations, for example to be scared, sad or happy |
| P3 | Questions related to a rating of 7 | ('A rigid delusional interpretation' means an interpretation that is based on a delusion and which is held on to, even if it is challenged by others, for example by asking about alternative interpretations.) |
| P4 | Definition | Excitement is reflected in behavior for example the patient can have a hard time sitting still (accelerated motor behavior) or the patient can react faster than usually if he/she sees something (heightened responsivity to stimuli). The patient can be hypervigilant, which means that the patient is over-attentive to the surroundings and that he/she reacts faster to stimuli in the room or constantly looks over the shoulder. Further the patient can have excessive mood lability which means that the patients mood is unstable and shifting, the patient can change from happy to irritated or angry in no time. |
| P4 | Questions related to a rating of 3 | ('Slightly pressured speech (in Danish there is one word for this) means an urge to speak a lot, the speech will often tend to be faster than usually.) |
| P4 | Questions related to a rating of 7 | ('Interpersonal interaction' means contact with other people.) |
| P5 | Definition | Delusions are beliefs that are unrealistic, unfounded and not shared by others. Examples of delusions are that the patient beliefs that he is Gods son or that he/she has special abilities, supernatural powers, or an unusually and unrealistic high IQ. |
| P5 | Questions related to a rating of 3 | ('Grandiose delusions' means unrealistic beliefs about extraordinary abilities, wealth, knowledge, fame, power or moral stature.) (* |
| P5 | Questions related to a rating of 6 | ('That the delusions have been acted upon' means that the patient has done something based on the delusion, for example that the patient has written letters to prominent people to disseminate his/her knowledge or that the patient has offered to heal other people.) |
| P5 | Questions related to a rating of 7 | ('Multiple' means more.) |
| P5 | Questions related to a rating of 7 | ('Bizarre' means that something is really odd or very deviant from the norm.) |
| P6 | Definition | ('Guardedness' (in the Danish translation: guarded behavior) means actions that the patient does in order to protect him-/herself (despite no apparent need to do this), it could be evident by the patient being reluctant or rejecting.) |
| P6 | Definition | ('Hypervigilant' means that a person is over-attentive to the surroundings and reacts fast to them.) |
| P6 | Questions related to a rating of 3 | ('A guarded attitude' is expressed by the patient being reluctant or rejecting in order to protect him-/herself |
| P6 | Questions related to a rating of 3 | ('Interpersonal relations' means relations between people, meaning relations between the patient and other people (. a therapist, family etc.) |
| P6 | Questions related to a rating of 6 | ('Pervasive delusions' means delusions that are comprehensive and that affect many aspects of the patients' life.) |
| P6 | Questions related to a rating of 6 | ('Systematized delusions' means that the delusions are put in system and are connected to each other |
| P7 | Definition | ('Verbal expressions' means the things that the patient says, while 'nonverbal expressions' means other forms of expression, for example body language and actions.) |
| P7 | Definition | ('Passive-aggressive behavior means behavior that is an indirect expression of anger, for example the patient can seem irritable or negative without expressing it directly.) |
| N2 | Definition | ('Life's events' in this context means events in everyday life.)(* |
| N2 | Questions related to a rating of 4 | ('Distanced emotionally' means to feel emotionally detached from other people.) |
| N2 | Questions related to a rating of 5 | ('Personal needs' means to take care of the most basic everyday functions such as personal hygiene, including bathing and brushing teeth regularly, and preparing and eating meals, cleaning up after oneself etc.) |
| N4 | Definition | ('Apathy' is a condition in which a person seems indifferent and uninterested in the milieu.) |
| N4 | Definition | ('Interpersonal relations' means relations between people.) |
| N4 | Questions related to a rating of 6 | ('Personal needs' means to take care of the most basic everyday functions such as personal hygiene, including bathing and brushing teeth regularly, and preparing and eating meals, cleaning up after oneself etc.) |
| G5 | Definition | ('A mannerism' is an odd, awkward and exaggerated way of moving or carrying out a certain action. For example the patient can come in to a room and bow in exaggerated way, stretching all the way to the floor or making an exaggerated movement with the arm.) |
| G5 | Definition | ('A disorganized or bizarre appearance' means that the patient looks and acts in a manner that is incoherent and purposeless or odd.) |
| G5 | Questions related to a rating of 3 | ('Rigidity' means that the patient freezes in a position.) |
| G5 | Questions related to a rating of 5 | ('Bizarre motoric rituals' are different kinds of movements or series of movements that are repeated in certain situations and which seem very unusual or odd.) |
| G5 | Questions related to a rating of 5 | ('Stereotyped movement' means a repetition of a certain movement, for example teeth grinding, rocking back and forward or rubbing the head. The movement does not seem to have a purpose or function for the patient in the situation.) |
| G6 | Questions related to a rating of 5 | ('Psychomotor retardation' means a diminution in the pace and/or the frequency of both speech and movements.) |
| G7 | Definition | ('Reduced body tone' means that the patient sags/collapses and does not sit or stand up straight.) |
| G7 | Definition | ('Diminished responsiveness to stimuli' means that the patient responds less than usually to the surroundings for example if someone says something.) |
| G7 | Questions related to a rating of 3 | ('Underproductive in conversation and gestures' means that the patient neither says much nor makes many gestures.) |
| G7 | Questions related to a rating of 4 | ('Long response latency' means that there is a long time gap from the moment a question is asked and until the patient reacts or replies to the question.) |
| G14 | Definition | ('Arbitrary' means random.) |
| G14 | Definition | ('Misdirected discharge of tension and emotions' means actions or emotions that are directed towards the 'wrong person'. It could be a burst of anger that is directed towards a person that is without blame and not the real target for the anger.) |
| G14 | Questions related to a rating of 4 | (Verbally abusive' means, that the patient says something that is abusive, for example insults.) |
| G14 | Questions related to a rating of 5 | ('P.r.n. medication' means medicine that is given or taken by need for example for anxiety or restlessness.) |
| G14 | Questions related to a rating of 6 | ('Hallucinatory commands' means hallucinations giving orders or instructions that the patient needs to do or follow, for example voices or visions telling or showing the patient to do a specific action.) |
| G16 | Questions related to a rating of 5 | ('Unstructured time' is the time where there is no prearranged activity.) |

**References**

1. Nielsen CM, Kølbæk P, Dines D, et al. Validation of ratings on the six-item Positive and Negative Syndrome Scale obtained via the Simplified Negative and Positive Symptoms Interview among outpatients with schizophrenia. *Journal of Psychopharmacology* Nov 2022;36(11):1208-1217.
